# Home is Where the Heterogeneity Is: Housing Facility-level Differences in the Gut Microbiome and Metabolic Phenotype Confound Arsenic Effects on Glucose Homeostasis in Male Mice

**DOI:** 10.64898/2026.08.03.742222

**Published:** 2026-08-03

**Authors:** Judy Malas, Lidan Zhao, Michael Landeche, Ashley M. Sidebottom, Jessica Little, Jarrad Hampton-Marcell, Robert M. Sargis

## Abstract

Inorganic arsenic (iAs) exposure is linked to impaired glucose homeostasis and type 2 diabetes, yet the magnitude and direction of reported effects vary substantially across studies and populations. The gut microbiome is both a target and a mediator of arsenic toxicity, suggesting that pre-exposure community composition may modulate the development of metabolic dysfunction. To test this, we conducted parallel 50 ppm iAs drinking-water exposures in male C57BL/6J mice at two animal facilities. Results were compared across facilities for metabolic phenotypes, hepatic arsenic levels, targeted and untargeted metabolomics, and shotgun metagenomics. Hepatic arsenic confirmed comparable exposure at both sites; however, the housing facility explained more variance than the iAs treatment group across every data layer. Baseline microbial communities and metabolic phenotypes at each institution differed, and this difference propagated into the iAs treatment effect. Critically, iAs exposure impaired glucose clearance at one site while trending toward improvement at the other. Facility explained 19 to 26% of variance in microbiome, bile acid, polar, and untargeted metabolite ordinations, while iAs treatment did not reach significance. A random forest classifier identified the facility with 96% cross-validated accuracy from 22 microbial species, whereas treatment classification did not exceed 67% accuracy. Functional metagenomic analyses revealed nearly 11,733 (63%) of genes were differentially abundant between facilities compared 139 with iAs treatment. Our results indicate that identical genetics and exposure may produce differential metabolic outcomes on different microbial backgrounds. Characterizing the baseline microbiome and metabolome is therefore critical both for identifying which individuals are most susceptible to the metabolic effects of arsenic exposure and for potentially reducing the risk of exposure through modulation of the gut microbiome.

## Full Text Availability

The license terms selected by the author(s) for this preprint version do not permit archiving in PMC. The full text is available from the preprint server.

